# “Smart Process” of Medical Innovation: The Synergism Based on Network and Physical Space

**DOI:** 10.3390/ijerph17113798

**Published:** 2020-05-27

**Authors:** Ailian Zhang, Mengmeng Pan

**Affiliations:** Business School, Jilin University, Changchun 130012, China; panmm19@mails.jlu.edu.cn

**Keywords:** medical innovation, public health, smart society, informatization, high-speed rail

## Abstract

Medical innovation has a profound impact on public health, and it is always of social concern to encourage innovation and enhance the value in health care delivery. Based on a sample of China’s listed firms in the medical industry from 2007 to 2018, this paper highlights the independent and mixed roles of informatization and high-speed rail in public medical innovation. The results show that informatization at network space and high-speed rail at physical space effectively promote the innovation of medical enterprises. In addition, “online” information technology and “offline” high-speed rail technology have a synergistic effect on medical innovation, especially in areas with a low level of innovation. The conclusion supports the positive significance of technology in the application of public health and proposes that the construction of smart society is very important to public health.

## 1. Introduction

Medical innovation has always exerted a profound impact on clinical practices, policymaking, and social expectations toward health care, and the importance of incentivizing innovation and elevating its value in health care delivery is of national focus [1,2,3,4,5,6]. However, nontechnical reasons seem to make medical innovation difficult to break through, and the imbalance of medical innovation between different regions is becoming increasingly prominent. The public health industry attempts to improve the quality of medicine by increasing cost-effectiveness and encouraging technological innovation [7]. At the same time, technological breakthrough is promised to fundamentally restructure the health business [8,9]. Therefore, based on the perspective of scientific and technological development, this paper explores the impact on medical innovation.

The development of technology is reflected in many aspects. This paper is mainly based on the dimension of information technology and high-speed rail (HSR) technology and the effect on medical innovation. The reason for choosing these two dimensions is that the evaluation of urban informatization level is considered as the basis of smart city evaluation [10], and the opening of high-speed rail makes cities become smart. The informatization index of this paper covers information technology more thoroughly, and the high-speed rail network covers Chinese cities more comprehensively. If we compare a “smart society” to an organism with vitality, data is the blood circulating in the organism, infrastructure is the nervous system of perception and transmission, big data platform is the circulatory system, urban governance is the sports system, industrial development is the digestive system, and people’s livelihood service is the respiratory system. Based on the “neural system” perspective of a smart society, this paper divides infrastructure into “online” infrastructure, namely network equipment, and “offline” infrastructure, namely physical equipment, to discuss the impact of informatization and high-speed rail technology on medical innovation. 

In recent years, Internet of things, cloud computing, big data, mobile internet, and other emerging information technologies have been widely used in various fields [11,12], most of which are focused on the fields of basic medicine and public health [13,14,15]. This is mainly because medical knowledge is an essential component of medical competency [16]. With the improvement of competitiveness, medical knowledge has emerged as the most strategically significant resource for the healthcare organization [17,18]. However, information technology can help enterprises not only acquire and absorb external knowledge [19], but also manage and share internal knowledge [20], so as to promote enterprise innovation [21]. Furthermore, the popularity of the internet allows the public to participate in health discussions, and the integration of the public in health and social research is essential for the advancement of research and development in health care [22,23,24]. In addition, although previous studies have specifically stated that technology is beneficial to the research and development of pharmaceutical companies [14,25,26], some articles raise questions about this subject. The unstructured data of internet contains too much insufficient information, and although the cost of knowledge acquisition is reduced, too much useless information increases the cost of knowledge identification [27]. Therefore, this paper has two advantages: first, the lack of emerging information technology data makes the relationship between technology and medical innovation controversial. This paper fills in the practice gap of the impact of informatization on medical innovation. Second, the above literature studied the impact of one aspect of information technology on pharmaceutical enterprises; however, the informatization index of this paper considers all aspects of information technology and more inclusively and accurately reflects the impact of informatization on the innovation ability of pharmaceutical enterprises.

Moreover, online communication cannot completely eliminate distance barriers, so distance is still an important factor in innovation development, while high-value added innovation activities often rely on face-to-face contact [28]. When the urban infrastructure is interconnected, the barriers of knowledge technology communication are eliminated, and innovation resources are no longer bound by fixed boundaries. The network characteristics of transportation infrastructure reduce the transportation cost and improve the spatial accessibility, which can realize the free flow of innovation elements and form innovation cross regional spillover [29]. As a new means of transportation, high-speed rail greatly reduces travel time and cost by improving city accessibility. The “space–time compression” effect of high-speed rail enables enterprises to share resources across greater geographical space and strengthen the speed and breadth of factor flow among enterprises [30,31]. In addition, the positive impact of high-speed rail on enterprise innovation is still controversial. For example, the opening of high-speed rail may cause the outflow of resource elements in the marginal areas, reduce the economic growth rate by bringing down fixed asset investment, labor migration and other forms, inhibit the innovation behavior of enterprises in cities along the line, and produce the “siphon effect” of innovation [32].

Previous studies about the impact of technology on enterprise innovation have mostly focused on the theoretical level, and many literatures affirm the important role of technology in enterprise innovation activities, believing that technology has a profound impact on enterprise innovation activities [33,34]. However, the influence of technology on enterprise innovation has industry heterogeneity [35]. From the perspective of information technology, Hellmanzik and Schmitz confirm that industries with high information density (finance, communication, etc.) are more sensitive to bilateral information flows on the Internet [36]. From the perspective of high-speed rail technology, the innovation effect of high-speed rail opening also has the influence of industry heterogeneity [37]. According to the new structural economics, industries with more comparative advantages may have more incentive effect of innovation because factor allocation conforms to regional factor endowment [38]. However, there are few empirical studies on the impact of technology on medical industry innovation. In 2013, the National Health and Family Planning Commission put forward the idea of building “smart medicine”, hoping to realize medical data sharing through the Internet. Medical experts can discuss treatment plans for difficult and miscellaneous diseases on the Internet, improve medical efficiency, and promote medical innovation [39]. Therefore, the impact of information technology and high-speed rail technology on the pharmaceutical industry cannot be ignored, technological breakthrough is promised to fundamentally restructure the health business [8,9]. 

The explosive growth of information and knowledge is beneficial to enterprise innovation, but the technology that brings explosive growth to information and knowledge requires cost. In addition, whether the combination of “online” technology and “offline” technology enhances or weakens the willingness of medical innovation needs further research. It is generally believed that there is complementarity between the impact of information technology and other technologies on enterprises [40]. However, according to resource-based theory, enterprises can be regarded as a collection of resources under the management framework [41]. If the internal management ability of medical enterprises is insufficient, the more resources medical enterprises invest, the more difficult it is to coordinate different resources, and the more difficult it is for medical enterprises to realize the synergy effect between different resources. When the management ability is relatively weak, it may even appear that the scheduling and use of resources interfere with each other, which is reflected as a negative interaction effect. There is a gap in the derivation and practice of the interactive influence of information and high-speed rail technology on medical innovation. This paper answers this question based on China’s technical data. Based on the “online” network space and the “offline” physical space dimensions, this paper studies the impact of technology on medical innovation. Furthermore, from the perspective of smart society, by introducing the interaction of information technology and high-speed rail, we can solve whether “online” technology and “offline” technology enhance or weaken each other’s positive effects on medical innovation.

China is a unique country in the field of medical and health care. First, this paper studies the innovation of medical enterprises in different cities, and the institutional and cultural differences between Chinese provinces are smaller than those in most other countries, meaning that the result bias caused by the heterogeneity of provinces will be reduced. Second, China has become the country with the longest operating mileage, the largest scale under construction, and the fastest development speed of high-speed railway in the world. After nearly 30 years of development, China has built the most modern and developed high-speed rail network in the world. Third, China is still a developing country, and it constantly tests feasible development methods. Because of the rapid application of information technology and high-speed rail technology in China’s medical field, the inspection caused by these two exogenous shocks is not easily disturbed by noise. The results of this paper supplement the gap in the existing research and verify the application effect of technology in medical innovation from the perspective of society. The results show that the information technology based on the network space dimension strengthens the innovation willingness of medical enterprises, and the high-speed rail technology based on the physical space dimension also promotes the medical innovation. In addition, informatization and high-speed rail have a synergism effect upon medical innovation. The results provide theoretical support for public health research and better reference for institution formulation. At the same time, it also provides a new research perspective for the imbalance development of public health in different regions.

In summary, this paper offers the following contributions. First, limited by data, most of the existing literature studies public health from the nontechnical level, and there are relatively few literatures exploring the innovation willingness of pharmaceutical enterprises from the technical level. This paper collects and arranges the data of high-speed railway manually. Its framework contributes to the extant literature by introducing the actual effect of informatization and high-speed rail construction on medical enterprises and enriches the research framework of medical innovation. Second, the existing literature mainly studies the impact of one aspect of information technology (Internet, online network health, etc.) on medical enterprises. This paper introduces the information index, considers the Internet, big data platform and other “online” technologies, studies the impact of information on medical innovation, and makes up for the relevant research on the impact of infrastructure construction on medical innovation. Third, it clearly distinguishes the impact of “online” technology and “offline” technology on medical innovation. Further, from the perspective of a smart society, we introduce the interaction between informatization and high-speed rail to discuss whether the complementarity between them can enhance or weaken the positive role of medical innovation, so as to fill the gap in the application of technology in medical innovation.

The structure of this paper is as follows: The second section contains the research background and hypotheses; the third section introduces the sample and data, variables, and research models; the fourth section expounds the empirical results, including descriptive statistics, regression results, and the research discussion; and the fifth contains the research conclusions.

## 2. Theory and Hypotheses

### 2.1. Basic Theory

The theory of innovation diffusion holds that innovation diffusion is influenced by the characteristics of innovation itself, communication channels, time, and social system [42]. The construction of a smart society has undoubtedly changed the diffusion environment of innovation and accelerated the diffusion speed of innovation. As the “nervous system” for perception and transmission of smart society, infrastructure plays an important role in strengthening the diffusion environment and accelerating the diffusion speed. From the perspective of communication channels, the development of information infrastructure (informatization) has broken through the traditional physical bottleneck of knowledge dissemination. People can use knowledge network to share and spread knowledge and information more quickly and conveniently and expand the sharing space. In view of communication time, the development of transportation infrastructure (high-speed rail) has broken the knowledge and information barriers, which makes knowledge not only used quickly but also verified effectively and shortens the sharing distance.

### 2.2. Hypotheses Development 

Based on the “nervous system” perspective of the smart society, informatization mainly improves the innovation diffusion environment and expands the information sharing space and promoted innovation activities [43]. The theory of information sharing and regeneration well explains the impact of informatization on innovation diffusion [44,45]. The theory of information sharing and regeneration means that the same information can be shared by many people, and the same information can be shared by people in different periods. It will not reduce the value because of the use of information. On the contrary, it can generate more information. The characteristic of information provides conditions for the sustainable development of a knowledge and information dependent society, while informatization strengthens the sharing and regeneration of information. As Solo said in his evaluation of Schumpeter’s theory of innovation, the source of new ideas and the realization process of ideas are two necessary conditions for innovation [46]. The development of information technology has fundamentally changed the environment for information exchange and distribution [47]. Pluye et al. study the effect of medical staff’s information use on patients’ interest perception [48]. Kim and Oh study the influence of website usability and aesthetics on the access of medical website users [49]. In addition, scholars in the field of medicine are also interested in doctors’ search for Internet information [39]. It can be seen that the impact of information on medical innovation has attracted more and more scholars’ interest.

In recent years, many studies have focused upon the impact of informatization on medical and public health innovation. The informatization mainly affects the medical innovation through three ways: First, the improvement of information level reduces the cost of information acquisition and improves the innovation ability of innovation subjects [50]. Knowledge and ideas are the most important catalysts in the innovation process [51,52]. Second, the improvement of information level can reduce the cost of information search and replication, and improve the efficiency of innovation resource allocation [53,54,55,56]. Third, the integration of internal production and operation of enterprises and information technology such as the Internet has a positive impact on innovation and export behavior [57,58]. Forman and Zeebroeck and Koellinger find that product innovation based on internet technology has a better impact on performance than product innovation based on traditional technology [59,60]. Based on this, we propose the first hypothesis:

**Hypothesis 1** **(H1).** *At the level of network space, informatization increases the medical innovation*.

Blum and Goldfarb believe that online communication cannot completely eliminate the impact of distance, so distance is still an important factor in economic development [28]. Based on the “nervous system” perspective of the smart society, the development of high-speed rail mainly saves innovation diffusion time and shortens the distance of information sharing, thus promoting innovation development [61,62]. The communication of innovation activities among pharmaceutical enterprises in different regions is a process from high potential energy to low potential energy. The path of innovation diffusion can be not only the physical space but also other factors. As an important carrier of mobile space, high-speed rail has a natural channel attribute and is the main body of innovation corridor in time and space. According to corridor model theory, the smoother the corridor, the better the effect of innovation diffusion. This result depends on the accessibility (travel time) and innovation potential energy difference. When the accessibility is high, if the potential energy difference of innovation between pharmaceutical enterprises in different regions is small, the effect of innovation diffusion is strong, otherwise, it is weak. When the accessibility is low, if the potential energy difference between pharmaceutical enterprises in different regions is small, the effect of innovation diffusion is weak; but if the potential energy difference between pharmaceutical enterprises in different regions is large, it is difficult to produce an innovation diffusion effect. The opening of high-speed rail improves the accessibility of cities and plays a leading role in the coordinated development of regional innovation [63]. When the accessibility is high, the innovation diffusion effect is strong. When the accessibility is low, the effect of innovation diffusion is weak. 

Under the effect of “space–time compression”, high-speed rail may reshape the spatial structure and economic distribution of factors by accelerating the circulation between cities [64]. There are two main ways of innovation incentive effect of high-speed rail: First, the opening of high-speed railway reduces the transportation cost, improves the passenger and freight volume of the city, and accelerates the flow of factors between regions, so as to improve the level of medical innovation. Second, the opening of high-speed rail increases the flow of people, logistics, and information between cities, generating expected benefits and improving the market potential [65,66]. Based on this, we propose the second hypothesis:

**Hypothesis 2** **(H2)**. *At the level of physical space, high-speed rail increases the medical innovation*.

When information technology and high-speed rail technology are combined, high-end elements such as people, logistics, and information flow are fully integrated and complementary. This process not only strengthens the innovation diffusion environment but also accelerates the innovation diffusion speed and has a synergistic effect on the medical innovation. According to the theory of coordination, coordination is a process in which multiple subsystems of a system cooperate with each other to form a unified whole and develop together. This process is often accompanied by the effect amplification, that is, “1 + 1 > 2” [67]. Based on “Synergism”, it is necessary to introduce the interaction of informatization and high-speed rail to conduct in-depth research on medical innovation.

Whether there is complementarity between information technology and high-speed rail technology is a key question. It is generally believed that there is complementarity between the impact of information technology and other technologies on enterprises [40]. This kind of complementarity is due to the supporting role of information technology in the use of other resources and activities of enterprises, so there is synergy between information technology and other related technologies [68]. This paper believes that the complementarity between information technology and high-speed rail technology leads to a positive interaction effect, that is, a synergism impact on the medical innovation. The evaluation of urban informatization level is considered as the basis of smart city evaluation [10], and the opening of high-speed rail connects the smart city. Therefore, on the premise that other dimensions remain unchanged, this paper constructs a simple smart society based on the dimensions of “online” network space and “offline” physical space, introduces the interaction of informatization and high-speed rail to study the synergistic effect on medical innovation.

**Hypothesis 3** **(H3).** *In the smart society, informatization and high-speed rail have a synergistic effect upon the medical innovation*.

## 3. Materials and Methods 

### 3.1. Sample and Data

This paper mainly intends to study whether the development of information technology and high-speed rail promotes the innovation of medical enterprises. According to the following two reasons, this paper chooses the listed firm as the subject of the research sample. First, there is a certain scale barrier to the application of technology. Public companies tend to be more mature and are more likely to break through this barrier, which can better verify the implementation effect of this test. Second, in view of data availability, listed companies must regularly disclose data in accordance with the provisions of the China Securities Regulatory Commission. Meanwhile, nonlisted companies do not have such a mandatory requirement, and the voluntary disclosure leads to the problem of sample selection bias. In summary, we chose China’s listed companies as the subjects for the initial research samples. In the screening process, we only keep the data of medical companies from 2007 to 2018. In addition, there are 34 provincial-level administrative regions in China. However, due to the lack of innovation data of medical enterprises in Neimenggu, Qinghai, Xinjiang, Ningxia, Xizang, Taiwan, Hong Kong, and Macao, the final data includes 156 medical enterprises in 26 provinces and cities, a total of 883 observations.

### 3.2. Variable Definitions

#### 3.2.1. Dependent Variable 

This paper aims to verify the impact of the application of information technology and high-speed rail technology on medical research and development (R&D). The main method to measure innovation ability of an enterprise are R&D investment, number of patents, and number of new product categories [49,69]. Among them, R&D investment reflects the input of enterprise innovation, and the number of patents and new product types measure the output of enterprise innovation. Foreign scholars have adopted patent number [35,70] and R&D investment [71,72,73], as a measure of innovation activities. But different from the developed countries, the number of patents and new products may not be suitable proxy variables in developing countries like China. First, many innovation activities of medical enterprises in developing countries are realized in the form of imitation, and this type of innovation activity cannot be displayed in the form of patents. Second, there are three types of patent applications for Chinese Enterprises: design patent, utility model patent, and invention patent. The first two cannot effectively measure the true independent innovation ability of enterprises, but only using invention patents cannot accurately measure the quality of innovation ability of enterprises. Third, a large proportion of patent applications of Chinese enterprises are filed by foreign-funded enterprises or foreign enterprises, especially invention patents. Fourth, due to the relative imperfection of intellectual property protection system in developing countries, enterprises’ innovation activities are not effectively protected. In this case, pharmaceutical enterprises lose the incentive to protect their own innovation interests through patent application. Fifth, compared with other industries, the innovation output of pharmaceutical enterprises takes a long time and requires a large amount of investment. If we use innovation output as a measure of medical innovation ability, we cannot accurately measure the change of enterprise innovation level.

The above facts show that for China’s pharmaceutical industry, using the number of patents or new products as a measure of innovation activities is not an effective measurement method. On the contrary, using R&D investment at the level of medical enterprises as a measure of innovation activities will not have the above problems. Furthermore, the development of information technology and the construction of high-speed rail provide sufficient information and knowledge support for pharmaceutical research and development, which accelerate the process of market demand, targeting, application experiment, and feedback improvement in pharmaceutical innovation. Innovation investment focuses on the willing perspective, and it is measured by the R&D expenditure disclosed in the financial statements divided by sales revenue. Because the data of R&D investment can be obtained in the database as early as 2007, the sample interval of this paper is from 2007 to 2018. R&D investment data of listed companies is obtained from CSMAR database.

#### 3.2.2. Independent Variables

This paper aims to verify the impact of information technology development and high-speed rail on medical R&D. The existing literature mostly uses broadband access, two-way link website, and other indicators to measure the information technology level of enterprises [74]. The informatization development index formulated by the National Bureau of statistics comprehensively evaluates the annual information development level of each province. The index includes internet related indicators. Informatization development index is a composite index composed of five elements reflecting informatization level (informatization infrastructure, industrial technology index, application consumption index, knowledge support index, and development effect index). For the convenience of research, this paper introduces informatization development index to measure the informatization development level of all provinces in China.

Based on the physical space dimension, the transportation facilities that can shorten the distance of knowledge sharing are airplanes, trains, etc. As one of the transportation systems, high-speed rail competes with other means of transportation in the choice of travel. In different travel distances, the competitiveness of high-speed rail and other modes of transportation is different. With the increase of distance, medium- and long-distance buses cannot compete with high-speed rail and aircraft in travel time [75]. But compared with aircraft, high-speed rail has the following advantages: First, the advantages of high-speed rail traffic volume are obvious. The cargo capacity of a high-speed rail car is equivalent to the load capacity of a Boeing aircraft, while a high-speed rail car has at least six or more cars. A high-speed rail train can carry more traffic than an aircraft. According to the development statistics bulletin of transportation industry issued by the Ministry of Transport in 2019, the passenger traffic volume of railway and civil aviation has achieved rapid growth. Passenger volume completed by railway in the whole year was 3660 million people, accounting for the proportion of the whole social business passenger traffic—20.8%; passenger volume completed by civil aviation was 660 million people, accounting for the proportion of the whole social business passenger traffic—3.8%. Second, compared with aircraft, high-speed rail has obvious advantages in price, convenience, and safety. Finally, according to statistics, the departure schedule of high-speed rail is more punctual than that of aircraft, and is less affected by natural factors such as weather. 

This paper uses high-speed rail as the representative variable of offline technology. In addition, because the information development index is calculated based on different provinces, the variable of high-speed railway is also measured by whether the high-speed railway is opened in different provinces. If the high-speed railway was opened in that province in a certain year, the value is 1, and if it was not opened, the value is 0. We collected 107 high-speed railways by hand and sorted out the provincial data of each high-speed railway route. Then we obtained the data of the opening year of high-speed railway in each province. HSR data was collected manually through the website of the National Railway Administration.

#### 3.2.3. Control Variables

In the research, the variables we controlled were not any independent variables that can cause changes in corporate innovation to make the results more accurate. Referring to the related literature on corporate innovation, this paper selected the following variables as the control variables. All control variables were obtained from the CSMAR database.

First, the basic characteristics of the medical company have an impact on the innovation situation. Therefore, we selected Size, Lev, IAR, and Tobin’s Q as control variables. Size refers to the size of a company, measured by the natural logarithm of the total assets. Lev (leverage) represents the company’s debt level, measured by dividing the company’s total liabilities by its total assets. IAR (intangible assets ratio) mainly measures the proportion of intangible assets of a company. It is the intangible assets of the current year divided by total assets at the end of the year. Tobin’s Q measures enterprise value, which is market price divided by replacement cost. 

Second, the operating conditions also promote or restrain the enterprises innovation. We selected ROA (return on assets) and growth. ROA is the return on total assets of the company during the year, representing the company’s profitability. Growth refers to the growth rate of the company’s total assets during the year and represents the company’s growth ability. 

Third, corporate governance has an important impact on corporate behavior, which is reflected in R&D strategy. Therefore, to verify the impact of informatization and high-speed rail on innovation in the model, the corporate governance variable should be controlled. In terms of this, we selected SOE (state-owned enterprise) to control. SOE represents the nature of ultimate corporate control. The business objectives and strategies of state-owned enterprises and non-state-owned enterprises are very different, and listed companies in China account for a high proportion of Chinese enterprises. 

Finally, in order to control for the characteristics of the firm that do not change with time and the annual characteristics that do not change with the province, we added province-fixed and year-fixed effects. Table 1 shows the specific variable definition.

### 3.3. Research Model

Based on the selected panel data, we construct the following model to verify the hypotheses. The explained variable is innovation representing the innovative actions of the enterprise. In the following equations, the explanatory variables are informatization, HSR, and informatization HSR, respectively. Among them, informatization HSR is introduced to study the interaction effect. When the interaction is positive, informatization and high-speed rail have a synergistic effect on medical innovation, and they will enhance each other’s positive effect on medical innovation. When the interaction is negative, informatization and high-speed rail have antagonistic effect on medical innovation, and they will weaken each other’s positive effect on medical innovation. The control variables included in the equation respectively cover the basic information, operation status, and governance of the company, as well as the fixed effect of the province and the fixed effect of the year. We use the OLS (ordinary least squares) to obtain the result. Under the condition that other possible interference factors are controlled, the effect of the informatization and high-speed rail on the company’s innovation is verified. The following models are used to test hypotheses 1, 2, and 3, respectively, which are the basic models for the regression of this paper. These variables are in the definition table.

(1) $$\begin{array}{cc} Innovation_{i,t} & =\alpha_{0}+\beta_{1}Informatization_{i,t}+\beta_{2}Size_{i,t}+\beta_{3}Lev_{i,t}+\beta_{4}IAR_{i,t}\\ & +\beta_{5}Tobin^{\prime }s\;Q_{i,t}+\beta_{6}ROA_{i,t}+\beta_{7}Growth_{i,t}+\beta_{8}SOE_{i,t}\\ & +Province\;fixed\;effect+Year\;fixed\;effect+\varepsilon_{i,t} \end{array}$$

(2) $$\begin{array}{cc} Innovation_{i,t} & =\alpha_{0}+\beta_{1}HSR_{i,t}+\beta_{2}Size_{i,t}+\beta_{3}Lev_{i,t}+\beta_{4}IAR_{i,t}+\beta_{5}Tobin^{\prime }s\;Q_{i,t}\\ & +\beta_{6}ROA_{i,t}+\beta_{7}Growth_{i,t}+\beta_{8}SOE_{i,t}+Province\;fixed\;effect\\ & +Year\;fixed\;effect+\varepsilon_{i,t} \end{array}$$

(3) $$\begin{array}{cc} Innovation_{i,t} & =\alpha_{0}+\beta_{1}Informatization_{i,t}+\beta_{2}HSR_{i,t}+\beta_{3}Informatization_{i,t}\times HSR_{i,t}\\ & +\beta_{4}Size_{i,t}+\beta_{5}Lev_{i,t}+\beta_{6}IAR_{i,t}+\beta_{7}Tobin^{\prime }s\;Q_{i,t}+\beta_{8}ROA_{i,t}\\ & +\beta_{9}Growth_{i,t}+\beta_{10}SOE_{i,t}+Province\;fixed\;effect\\ & +Year\;fixed\;effect+\varepsilon_{i,t} \end{array}$$

In the above cross-section model, i stands for firm i and t stands for accounting period t. $\alpha_{0}$ is the intercept term of three equations, and β is the coefficient value of each variable used to check the degree of correlation between independent and dependent variables. The year-fixed effect controls the interference of time series, and the province-fixed effect controls the influence of regional differences. ε is the random perturbation term in the panel equation. In model 1, if $\beta_{1}$ > 0, H1 is verified. In model 2, if $\beta_{1}$ > 0, H2 is verified. In model 3, if $\beta_{3}$ > 0, which represents informatization and high-speed rail have a synergistic effect on medical innovation, H3 is verified. Empirical regression is carried out according to the above model. The empirical results are expounded in the following part.

## 4. Results

### 4.1. Descriptive Statistical Analysis 

Table 2 shows the results of the descriptive statistics. As can be seen from the second column, the sample number of all variables is 883. The mean of innovation is larger than the median, indicating that the innovation is the key to competition in pharmaceutical industry. But, how to strengthen medical innovation needs further study. The variance of informatization is 0.517, meaning that the level of informatization among different provinces varies greatly. However, the median of informatization (1.831) is far greater than the minimum value (0.508), and is close to the maximum value (1.998). The result means that the level of informatization has increased explosively in recent years, and the growth rate of informatization is very fast. In addition, among the control variables, only the variance of Tobin’s Q (2.318) is greater than 1, which also indicates that the enterprise value of different enterprises in the pharmaceutical industry is very different. The mean of SOE is 0.285, which shows that pharmaceutical enterprises are mostly non-state-owned, while state-owned enterprises are few.

Due to the different level of informatization and the opening of high-speed rail in different provinces, this paper takes the average of the innovation data of all pharmaceutical enterprises in each province to observe the province heterogeneity of medical innovation. We calculate the pharmaceutical innovation ranking of 26 provinces, autonomous regions, and municipalities in China. 

**Figure 1:**
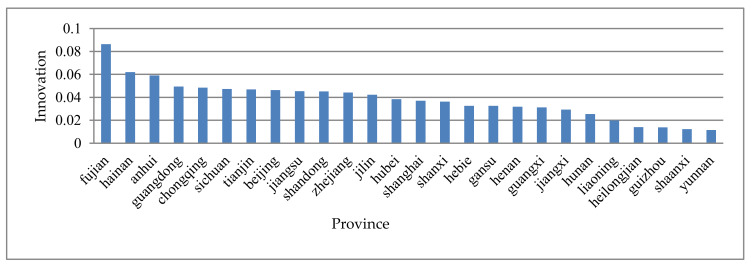
Innovation ranking of 26 provinces. Weighted average of innovation data of all medical enterprises in the province during the sample period is the innovation level of the province. This paper excludes the data of Neimenggu, Qinghai, Xinjiang, Ningxia, Xizang, Taiwan, Hong Kong, and Macao.

Figure 1 shows the ranking of R&D investment of pharmaceutical enterprises in different provinces. On the whole, China’s regional innovation ability has significantly enhanced, and the regional innovation pattern with different characteristics has basically formed, but the innovation level of each region is unbalanced. From Figure 1, we can see that in the period 2007–2018, the highest average medical innovation in 26 provinces of China is Fujian (0.086) and the lowest is Yunnan (0.011). Fujian’s willingness medical innovate is almost eight times that of Yunnan. In addition, the medical innovation hospitals in East China are relatively strong (except Jiangxi), while those in central and Western China and Northeast China are generally low (except Chongqing and Sichuan). Fujian and Guangdong in South China are far ahead in medical innovation, while Guangxi has a large space for improvement. Although China’s regional innovation capacity has significantly improved, in practice, there are still imbalances and inadequacies in regional innovation, so there is still room to improve the level of medical innovation in different regions. The root cause of this phenomenon calls for further study.

### 4.2. Regression Analysis

The development of infrastructure is indispensable for the construction of smart cities, and the evaluation of urban informatization level is considered as a foundation of smart city evaluation [10], and the opening of high-speed rail makes cities become smart. Therefore, in this paper, infrastructure is divided into “online” infrastructure and “offline” infrastructure. Based on the perspective of informatization and high-speed rail, we study the “smart process” of medical innovation. Table 3 shows the results of the basic regressions. In each set of validations, columns (1), (2), and (3) verify hypothesis (1), (2), and (3), respectively. It can be seen from the results in the table that informatization, HSR, and informatization × HSR all are significantly positively correlated with innovation. Because of the difference of informatization development level and high-speed rail opening in different provinces, we add the province-fixed effect to the regression in order to better control the heterogeneity of each province. At the same time, to control year-to-year variability, we add the year-fixed effect to the regression. Considering the economic implications, we can see from the table that, in the case that the control environment is taken into account, for every informatization unit added, medical innovation increases by 0.042, which proves H1. Similarly, when the provincial high-speed railway opens, medical innovation increases by 0.014 units, which proves H2. The improvement of provincial informatization level and the opening of high-speed railway between provinces enhance the innovation willingness of medical enterprises. At the same time, the interaction between informatization and high-speed rail has a significant positive effect on medical innovation, indicating that informatization and high-speed rail enhance each other’s positive effect on medical innovation. Therefore, based on the perspective of a smart society, “online” network space and “offline” physical space have a synergistic effect on medical innovation (0.015), which proves H3.

In terms of control variables, both Lev and SOE are significantly negative at the 1% level, while growth is significantly negative at the 5% level, indicating a higher leverage ratio, higher growth of medical enterprises, and lower innovation willingness. Because of the higher leverage ratio and higher growth, the medical enterprises do not have rich cash flow for R&D. In addition, compared with non-state-owned medical enterprises, state-owned medical enterprises are less willing to innovate. Tobin’s Q and IAR are both significantly positive at the level of 1%, indicating that the higher the enterprise value is, the greater the proportion of intangible assets, and the higher the willingness of medical innovation.

### 4.3. Regional Medical Innovation Imbalance 

It can be seen from Figure 1 that the willingness of medical innovation varies greatly in different provinces of China. In fact, this problem does not only happen in China, and the problem of regional innovation imbalance exists around the world. Therefore, based on the technology perspective, we further divide medical enterprises into high innovation willingness group and low innovation willingness group to explore how to solve the regional innovation imbalance. First, we define the innovation level of the enterprise. When the innovation level of the enterprise is larger than the average innovation, we define it as high, and when the innovation level of the enterprise is smaller than the average innovation, we define it as low. The heterogeneity test results for enterprise innovation are shown in Table 4.

From the perspective of information technology, the positive impact of informatization on the high innovation group (0.044) is far greater than that of the low innovation group (0.009). Similarly, from the perspective of high-speed rail technology, the positive impact of high-speed rail on the high innovation group (0.023) is far greater than that of the low innovation group (0.005). The results show that the higher the degree of innovation of medical enterprises, the more vulnerable they are to the impact of information technology and high-speed rail, thus increasing R&D investment. However, based on the perspective of a smart society, the interaction between informatization and high-speed rail has a significant positive impact on the low innovation group (0.008), while the impact on the high innovation group is not significant. Therefore, promoting the development of smart society will help to solve the imbalance and insufficiency of medical innovation.

## 5. Discussion

Based on the perspective of informatization and high-speed rail, this paper studies the impact of both two on medical innovation. Furthermore, from the perspective of a smart society, we introduce the interaction between information technology and high-speed rail technology to expound whether the combination of “online” technology and “offline” technology has synergistic or antagonistic effect on medical innovation.

From the perspective of information technology, the development of information technology has spatial and temporal heterogeneity, that is, different provinces have different levels of information technology at different times. The empirical results show that the higher the level of information technology, the greater the willingness of medical innovation. So, the development of information technology is one of the key factors to promote medical innovation. In summary, the new round of information technology revolution has changed the traditional way of medical innovation. The new generation of information technology revolution and its innovative application represented by big data, cloud computing, and Internet of things are strengthening the willingness of medical innovation.

From the perspective of physical space, the opening of high-speed rail connects provinces and increases the circulation of medical talents, medical equipment, and medical knowledge in each province, thus promoting medical innovation. Innovation is mostly carried out at the government level or under the guidance of the government, and the main position of enterprises innovation is not prominent. The development of high-speed rail technology allows innovation resources to fully flow and be shared in the region. The empirical results show that the opening of high-speed rail significantly enhances the willingness of pharmaceutical enterprises to innovate. In addition to the virtual network space, the shortening of physical distance is also conducive to the dissemination of information or knowledge to promote enterprise innovation.

Information technology expands the knowledge sharing space, and high-speed rail technology shortens the distance of knowledge sharing. With the combination of “online” and “offline”, “expansion of space”, and “shortening of distance”, the interaction between information technology and high-speed rail technology has a synergistic effect on medical innovation. The empirical results show that the development of a smart society is conducive to medical innovation, especially in areas with weak innovation level. Because the level of medical innovation in each province is unbalanced and the gap between “North South” and “East West” is large, the combination of “online” technology and “offline” technology shortens the “polarization” gap of innovation level, and brings a new research perspective to solve the problem of unbalanced and inadequate innovation development.

## 6. Conclusions

In terms of how to improve the level of public health effectively, existing research has reported on very comprehensive studies and mainly focuses on the importance of nontechnical perspective. From the perspective of the impact of science and technology, this paper introduces two exogenous events of informatization and high-speed rail to study the disruptive changes brought by technological shock to the pharmaceutical industry. The results show that both the improvement of information technology and the opening of high-speed rail improve the innovation willingness of provincial pharmaceutical enterprises. Furthermore, the interaction of information technology and high-speed rail technology on medical innovation is positive.

There are two theoretical meanings in this paper. First, based on the innovation diffusion theory, this paper studies the impact of intelligent infrastructure improvement on innovation diffusion environment and innovation diffusion channels from the perspective of a smart society, which is helpful for the expansion of this theory. Second, based on the theory of coordination, this paper introduces the interaction of informatization and high-speed rail to conduct in-depth research on medical innovation, which fills the gap in the relevant research on the impact of infrastructure construction on medical innovation. Our research has numerous practical implications. First, our research provides advice for how to further improve public health. Innovation is inseparable from the support of the ecological environment such as personnel, resources, and market. The opening of high-speed rail has accelerated the flow of people, logistics, and information. Therefore, the innovative ecological environment has an important impact on the innovation efficiency and innovation ability of pharmaceutical enterprises. Second, in solving the unbalanced development of regional pharmaceutical innovation, this paper offers management suggestions for deepening the reform of the scientific and technological system and further improving the medical innovation system. The wide application of new generation information technology has broken the limitation of innovation network limited by geography. A series of changes indicate that the demand of medical innovation for the reform of scientific and technological organization system is increasingly strong. Third, the results of our research also provide guidance for focusing on the influence of smart society on the medical field. Now, the pace of scientific and technological innovation is obviously faster than that of social innovation. People’s desire for a better life highlights the shortcoming of social public service. It is suggested to further promote the integrated development of scientific and technological innovation and social services, attach importance to and promote the technological innovation work in the field of social public services such as medical care and pension, and improve the level of social public health.

The current study also has several limitations that should be addressed in future research. First, the impact of information technology and high-speed rail technology on public health is not only limited to the micro level of this study but also based on the meso level of industry and macro level of society perspective. Second, the smart society is not based on one or two dimensions, but multi dimensions. In the design of this paper, assuming that other dimensions remain the same, we discuss the impact on medical innovation only from the infrastructure dimension of smart society. Future research should consider the construction of other dimensions.

## Figures and Tables

**Table 1:** Variable definitions.

Variable	Variable Definition
**Dependent Variable**	
Innovation	This variable represents the innovative actions of the enterprise. It is the R&D (research and development) expenditures/sales revenue.
**Independent Variables**	
Informatization	This variable is measured by informatization development index (IDI).
HSR	High-speed rail is a dummy variable. The year before the opening of HSR in each province is 0, and the year after the opening of high-speed rail in each province is 1.
**Control Variables**	
Size	This variable is used to measure the size of a company. It is calculated by the natural log of the firm’s total assets.
Lev	Lev (leverage) is used to measure a company’s debt level. It is calculated by the ratio of total liabilities to total assets.
IAR	IAR (intangible assets ratio) measures the proportion of intangible assets of a company. It is the intangible assets of the current year/ total assets.
Tobin’s Q	Tobin’s Q measures enterprise value, which is market price/ replacement cost.
Growth	This variable measures the growth status of the company. Growth = (this year’s sales revenue minus last year’s sales revenue) × 100%/ last year’s sales revenue.
ROA	ROA (return on assets) mainly measures the profitability of the company. It is the net profit of the current year/total assets at the end of the year.
SOE	SOE (state-owned enterprise) is a dummy variable that distinguishes property rights. It equals one if the firm is state owned, and zero otherwise.
Year-fixed effect	Year dummy variable, controlling the interference factors in time series.
Province-fixed effect	Province dummy variable, controlling the interference factors in cross section.

Notes: This variable definition table contains all of the variables in the baseline regression.

**Table 2:** Descriptive statistics.

Variable	Obs.	Mean	Max	Min	Median	Std. Dev.
Innovation	883	0.042	0.283	0.000	0.037	0.030
Informatization	883	1.530	1.998	0.508	1.831	0.517
HSR	883	0.904	1.000	0.000	1.000	0.295
Size	883	9.467	10.849	8.400	9.456	0.426
Lev	883	0.321	1.163	0.008	0.301	0.192
IAR	883	0.051	0.326	0.000	0.042	0.038
Tobin’s Q	883	3.495	15.065	0.801	2.766	2.318
ROA	883	0.071	0.494	−0.367	0.063	0.068
Growth	883	0.131	0.935	−1.393	0.143	0.215
SOE	883	0.285	1.000	0.000	0.000	0.452

Notes: Obs. is the sample observed number. Mean is the mean. Max is the maximum. Min is the minimum. Median is the value in the middle of the sample ascending order. Std. Dev. is the standard deviation. Sample range from 2007 to 2018.

**Table 3:** Regression analysis table.

Variable	Innovation		
	(1)	(2)	(3)
Informatization	0.042 *** (3.849)		0.022 (1.611)
HSR		0.014 *** (3.079)	−0.005 (−0.483)
Informatization × HSR			0.015 * (1.650)
Size	0.001 (0.400)	0.002 (0.792)	0.002 (0.536)
Lev	−0.024 *** (−4.142)	−0.026 *** (−4.422)	−0.025 *** (−4.169)
IAR	0.082 *** (3.195)	0.066 *** (2.600)	0.077 *** (3.036)
Tobin’s Q	0.003 *** (4.855)	0.003 *** (5.069)	0.003 *** (5.014)
ROA	−0.025 (−1.390)	−0.019 (−1.049)	−0.023 (−1.297)
Growth	−0.010 ** (−2.226)	−0.010 ** (−2.139)	−0.010 ** (−2.320)
SOE	−0.010 *** (−4.500)	−0.010 *** (−4.371)	−0.010 *** (−4.473)
Constant	−0.032 (−1.041)	0.009 (0.345)	−0.023 (−0.750)
Year	yes	yes	yes
Province	yes	yes	yes
Observations	883	883	883
R2	0.127	0.121	0.134

Notes: The superscripts ***, **, and * indicate significance at the 1%, 5%, and 10% levels, respectively.

**Table 4:** Regression analysis table.

Variable	Innovation					
	(1)		(2)		(3)	
	High	Low	High	Low	High	Low
Informatization	0.044 ** (2.352)	0.009 * (1.718)			0.018 (0.583)	−0.001 (−0.059)
HSR			0.023 ** (2.362)	0.005 ** (2.438)	0.002 (0.072)	−0.004 (−0.784)
Informatization × HSR					0.020 (0.788)	0.008 * (1.918)
Size	0.003 (0.586)	−0.006 *** (−3.977)	0.004 (0.839)	−0.006 *** (−3.801)	0.002 (0.522)	−0.006 *** (−3.758)
Lev	−0.001 (−0.010)	0.011 *** (−3.679)	0.002 (0.226)	−0.012 *** (−3.963)	0.002 (0.201)	−0.011 *** (−3.830)
IAR	0.119 ** (2.482)	0.012 (0.940)	0.107 *** (2.256)	0.007 (0.590)	0.117 ** (2.436)	0.009 (0.750)
ROA	−0.004 (−0.136)	0.028 *** (2.909)	0.005 (0.172)	0.029 *** (3.037)	−0.002 (−0.081)	0.029 *** (3.010)
Growth	−0.014 * (−1.677)	−0.001 (−0.133)	−0.014 * (−1.712)	−0.001 (−0.148)	−0.015 * (−1.839)	−0.001 (−0.204)
Tobin’s Q	0.004 *** (5.213)	−0.001 *** (−3.497)	0.004 *** (5.328)	−0.001 *** (−3.272)	0.004 *** (5.334)	−0.001 *** (−3.325)
SOE	0.002 (0.506)	−0.003 ** (−2.309)	0.002 (0.362)	−0.002 ** (−2.323)	0.001 (0.280)	−0.003 ** (−2.290)
Constant	−0.049 (−1.044)	0.074 *** (4.514)	−0.010 (−0.239)	0.081 *** (5.455)	−0.037 (−0.743)	0.078 *** (4.706)
Year	yes	yes	yes	yes	yes	yes
Province	yes	yes	yes	yes	yes	yes
Observations	373	510	373	510	373	510
R2	0.125	0.136	0.125	0.141	0.135	0.150

Notes: The superscripts ***, **, and * indicate significance at the 1%, 5%, and 10% levels, respectively.
